# Dietary patterns and Alzheimer's disease: East-west perspectives and future intervention strategies

**DOI:** 10.1016/j.tjpad.2026.100636

**Published:** 2026-07-08

**Authors:** Wanlu Jiang, Yijia Lin, Qihao Guo, Ya Miao

**Affiliations:** Department of Geriatrics, Shanghai Sixth People's Hospital Affiliated to Shanghai Jiao Tong University School of Medicine, 600 Yishan Rd., Xuhui District, Shanghai, 200233, China

**Keywords:** Alzheimer's disease, Diet, Cognition, Eastern dietary patterns, Precision nutrition

## Abstract

Among various modifiable risk factors, dietary patterns (DPs), as a holistic lifestyle intervention, have become a focus of current research due to their protective effects on cognitive health. Classic Western DPs, such as the Mediterranean diet (MedDiet), have been widely confirmed to effectively improve cognitive function, thereby reducing the risk of Alzheimer's disease (AD). However, existing evidence mainly concentrates on Western populations and their DPs, including the MODERN (Machine learning-assisted Optimizing Dietary intERvention against demeNtia risk) diet optimized using machine learning. Given the significant differences in food types, dietary habits, and cooking methods among Asian populations, research on localized DPs optimized for cognitive health in Asian populations remains insufficient. In this context, the team at the Department of Geriatrics, Shanghai Sixth People's Hospital Affiliated to Shanghai Jiao Tong University School of Medicine has taken the lead in systematically defining the Shanghai Cognitive Diet Pattern (SCDP). This review aims to comprehensively outline the core features and potential biological mechanisms relevant to AD in both classic Western DPs and the emerging East Asian DP. Subsequently, this review will systematically compare Eastern and Western DPs. In conclusion, this review proposes shifting​ dietary strategies from population-level adaptation to individual precision, in conjunction with multimodal lifestyle management, and offers novel strategies for the prevention and management of AD.

## Highlights

• Compares five Western dietary patterns, including the MODERN (Machine learning-assisted Optimizing Dietary intERvention against demeNtia risk) diet optimized using machine learning.
• Introduces the Shanghai Cognitive Diet Pattern (SCDP) based on Eastern populations.
• Analyzes the similarities and differences between Eastern and Western dietary patterns.
• Proposes shifting dietary strategies from population-level adaptation to individual precision, in conjunction with multimodal lifestyle management.

## 1 Introduction

As the global population ages at an accelerating pace, the number of dementia cases worldwide is surging at an alarming rate. Projections indicate that the global dementia population is anticipated to increase from 57.4 million in 2019 to 152.8 million in 2050 [1]. Alzheimer's disease (AD) is the leading cause of dementia, contributing to approximately 60% to 70% of all cases [2]. AD is a neurodegenerative disorder defined by progressive cognitive deterioration. Beyond compromising patient quality of life, AD imposes immense emotional and financial burdens on families. In 2023, the total societal cost of dementia worldwide reached $614.8 billion, with direct healthcare expenditures alone projected to surge to $1.6 trillion by 2050 [3].

Currently, AD remains incurable. While cholinesterase inhibitors (e.g., donepezil) and NMDA receptor antagonists (e.g., memantine) are the primary medications used for AD, their effectiveness is limited. These medications provide only temporary relief from cognitive symptoms but fail to delay the disease's overall progression. Recently, anti-β-amyloid antibody therapies targeting the core pathological mechanisms of AD, such as Aducanumab, have shown some efficacy. However, their clinical application has been limited due to controversies over therapeutic benefits and severe side effects, among which amyloid-related imaging abnormalities are frequently observed [4]. Given the current limitations of drug treatments for AD, the focus of intervention is transitioning from traditional treatment toward early risk management.

As the primary type of dementia, the risk factor profile of AD largely reflects that of dementia in general, highlighting risk factor management as a crucial strategy for early prevention. Among modifiable risk factors, dietary patterns (DPs) offer distinct advantages due to their multi-target regulatory effects and long-term safety. Classic Western DPs such as the Mediterranean diet (the MedDiet), the Dietary Approaches to Stop Hypertension (DASH) diet, and the Mediterranean-DASH Intervention for Neurodegenerative Delay (MIND) diet have been widely confirmed to effectively reduce the risk of AD. However, most existing evidence focuses primarily on Western populations. Substantial differences exist between Asian dietary habits and those of other regions, especially in food types and cooking methods. Therefore, localized DP research tailored to Asian cognitive health is currently lacking. Against this backdrop, the Geriatrics study group at Shanghai Sixth People's Hospital Affiliated to Shanghai Jiao Tong University first systematically defined the Shanghai Cognitive Dietary Pattern (SCDP). This review aims to systematically outline and compare the core features and potential biological mechanisms in AD for these classic Western DPs and emerging East Asian regional DPs. Ultimately, this analysis highlights the necessity of shifting dietary strategies from population-level adaptation to individual precision, synergized with multimodal lifestyle interventions, and provides a theoretical foundation for promoting the precise prevention paradigm of AD.

## 2 The relationship between DPs and AD

Individual food consumption occurs within a complex, interconnected system rather than in isolation. Reflecting this complexity, nutritional research has shifted from individual nutrients toward a comprehensive approach analyzing overall DPs [5]. This shift more accurately reflects people's daily dietary habits. Research indicates that the neuroprotective effects generated by integrating multiple foods and nutrients into comprehensive DPs are generally considered superior to supplementation with single nutrients [5,6]. DPs can be categorized in various ways: For instance, as protective or harmful based on their health impacts, or as traditional or emerging depending on their historical development. In this paper, adopting a geographic-cultural perspective, we mainly divide these patterns into two broad categories: Western DPs and Eastern DPs.

### 2.1 Western DPs

Essential dietary components include protective foods, such as fruits, vegetables, and whole grains. Their consumption is universally advocated and represents a fundamental commonality among healthy DPs. However, these patterns exhibit structural differences in the composition of moderately consumed and restricted components. We have therefore systematically summarized the detailed comparison of key food components across these five DPs in Table 1.

**Table 1:** Comparison between western DPs.

MedDiet, the Mediterranean diet; DASH, Dietary Approaches to Stop Hypertension; MIND, Mediterranean-DASH Intervention for Neurodegenerative Delay; WD, the Western diet; MODERN, Machine learning-assisted Optimizing Dietary intERvention against demeNtia risk.

#### 2.1.1 MedDiet

The MedDiet, well-known as a healthy DP, exerts positive effects on the cognitive performance of individuals with AD. This DP emphasizes abundant intake of plant-based foods, fish, and olive oil. It recommends moderate consumption of red meat and dairy products, while permitting limited amounts of wine [7]. The health benefits of the MedDiet extend beyond food intake to encompass holistic lifestyle habits, including seasonal food selection and moderate drinking. When integrated within a broader social and behavioral context, these elements collectively form a sustainable model for healthy living [8].

The cognitive benefits of the MedDiet are supported by multiple lines of evidence. First, a UK Biobank study demonstrated that higher adherence to the MedDiet is associated with a reduced risk of developing dementia​. This association is independent of genetic risk factors, emphasizing diet as an important modifiable risk factor in the primary prevention of dementia [9]. Furthermore, a cohort study of middle-aged and older adults in the United States further reinforced this association. Individuals with higher scores on the alternative Mediterranean diet exhibited significantly lower AD mortality and a reduced incidence of psychometrically defined mild cognitive impairment [10]. More specifically, greater adherence to the MedDiet showed a significant correlation with improved overall cognitive function, preserved memory, and slower cognitive decline in older adults [11]. Similarly, supporting evidence also emerged from a longitudinal study of older adults in Greece, the birthplace of the MedDiet. This research demonstrated that a robust association between greater adherence to the MedDiet and a reduced incidence of dementia, as well as enhanced multidimensional cognitive performance, including memory, language, visuospatial perception, and overall cognitive function. The study also emphasizes that among the MedDiet components, fish and non-refined cereals serve as independent predictors, significantly correlating with reduced dementia risk and higher cognitive scores, respectively [12].

Mechanistically, the neuroprotective potential of the MedDiet is not attributable to a single component but rather to the synergistic interactions of multiple nutrients. A primary pathway involves its potent anti-inflammatory and antioxidant properties, as well as its capacity to modulate the gut-brain axis. For example, as a major dietary component of the MedDiet, fish is rich in long-chain omega-3 polyunsaturated fatty acids, especially eicosapentaenoic acid and docosahexaenoic acid [13]. These fatty acids exhibit potent anti-inflammatory properties [14]. Notably, studies indicate a significant inverse correlation between circulating omega-3 fatty acid levels and the risk of dementia [15,16]. Moreover, flavonoids from fruits, vegetables, and red wine exhibit robust anti-inflammatory effects by targeting and inhibiting inflammatory signaling pathways such as NF-κB. Furthermore, the MedDiet also provides a wide range of antioxidants, including vitamins C and E, and plant-based flavonoids [17]. These small-molecule antioxidants effectively scavenge free radicals, thereby mitigating oxidative stress-induced damage in neuronal cells. Another important feature of the MedDiet is its high fiber content from whole grains, vegetables, and fruits. The gut microbiota ferments this dietary fiber to produce short-chain fatty acids, such as butyrate [18]. These short-chain fatty acids not only exert systemic anti-inflammatory effects but also positively influence brain health via the gut-brain axis [19]. Additionally, a more direct mechanism may lie in its capacity to counteract the core pathology of AD. For example, a long-term study found that adherence to the MedDiet, especially with high fruit consumption, was linked to reduced amyloid-beta (Aβ) plaque deposition in the brains of cognitively healthy adults, which constitutes a key pathological hallmark of AD [20]. In summary, the MedDiet demonstrates significant potential to delay cognitive decline and prevent AD via the synergistic interplay of multiple mechanisms, specifically through improving the cerebral microenvironment, modulating inflammatory responses, and potentially targeting directly in Aβ pathology.

#### 2.1.2 DASH diet

The DASH diet was initially developed primarily for the prevention and management of hypertension. Its dietary structure shares similarities with the MedDiet, emphasizing abundant intake of plant-based foods, including fruits, vegetables, and whole grains, alongside low-fat dairy products. Moreover, the DASH diet is rich in potassium, magnesium, and calcium, while restricting saturated fat, total fat, cholesterol, and refined sugar [21]. It also imposes stricter limits on sodium intake, sugary beverages, and red meat, and advises against​ alcohol consumption [7].

In recent years, the DASH diet's potential to protect brain health has garnered considerable attention. Several studies indicate that greater adherence to the DASH diet can significantly reduce the risk of developing AD [22]. However, evidence on whether the DASH diet can effectively slow down cognitive decline in AD patients remains inconsistent. For instance, in a prospective cohort study involving 16,144 older women, Berendsen et al. demonstrated that long-term adherence to the DASH diet was strongly associated with better cognitive function, including overall cognition and verbal memory. Vegetables, nuts, and legumes were recognized as primary dietary components contributing to these cognitive benefits. Despite this, the study did not observe a notable attenuation of cognitive decline over a six-year follow-up [23]. Conversely, Tangney et al. observed that adherence to the DASH diet was significantly correlated with a reduced rate of cognitive decline in a population with an average age of 81.5 years [24]. This suggests that the DASH diet's impact on cognitive function may vary depending on the age and health condition of the individuals involved in the study. Furthermore, a comparative study between the MedDiet and DASH diets revealed that adherence to the DASH diet was linked to superior baseline cognitive performance and a slower rate of cognitive decline. Baseline performance was defined as the global cognitive composite score at study entry, calculated based on assessments of immediate and delayed recall, serial 7 s subtraction, and backward counting tasks. Compared to the MedDiet, the DASH diet had a more robust and statistically significant association with both baseline cognitive levels and cognitive change rates [25]. This indicates that, within this cohort, adherence to the DASH diet may be more robustly linked to the preservation of overall cognitive function.

From a mechanistic perspective, the neuroprotective benefits of the DASH diet on cognitive function are complex and multifaceted. On one hand, the DASH diet exhibits anti-inflammatory properties and has been shown to effectively improve circulating inflammatory biomarkers. This effect may be attributable to its high content of fiber, antioxidants, magnesium, and calcium, coupled with its low glycemic index characteristics, all of which work together to suppress inflammatory processes [26]. For instance, a meta-analysis of randomized trials found that adherence to the DASH diet significantly reduced serum high-sensitivity C-reactive protein levels compared to a usual diet [26]. On the other hand, similar to the MedDiet, the DASH diet provides abundant anti-inflammatory and antioxidant compounds from fruits, vegetables, legumes, and nuts, which may decrease cerebral oxidative stress, promote neurogenesis, and enhance neuronal connections. Another key advantage of the DASH diet is its ability to lower high blood pressure, which is a major risk factor for AD [27].

#### 2.1.3 MIND diet

The MIND diet integrates elements from both the MedDiet and DASH DPs to boost their positive effects. It encourages ten food categories, including leafy green vegetables, other vegetables (e.g., broccoli, carrots, peppers, tomatoes, and onions), berries, nuts, olive oil, whole grains, fish, poultry, legumes, and moderate amounts of red wine, while restricting five categories, such as red meat, butter, cheese, desserts, and fried foods.

Numerous studies confirm that high adherence to the MIND diet is closely correlated with superior cognitive function and a slower rate of cognitive decline, underscoring the important role of this DP in maintaining cognitive resilience [28]. A prospective cohort study by Morris et al., involving 960 older adults, found that participants with the highest adherence experienced significantly slower cognitive decline compared to those with the lowest adherence. This difference equated to a cognitive age 7.5 years younger and revealed a stronger association with cognitive decline than adherence to either the MedDiet or the DASH diet [29]. Another major advantage of the MIND diet is its neuroprotective properties, which are evident even at lower levels of adherence. A 4.5-year follow-up study involving 923 participants found that individuals with moderate adherence to the MIND diet experienced a 35% reduction in the risk of developing AD compared to those with the lowest adherence, whereas strict adherence reduced the risk by as much as 53%. In contrast, the MedDiet and DASH diets exhibited similar protective effects only among individuals with the highest adherence [30]. These results imply that strict perfection is not required to obtain benefits from the MIND diet, thereby enhancing its feasibility and long-term applicability for promotion among the general population.

Unlike the MedDiet, which was originally designed to promote cardiovascular health, and the DASH diet, which was initially developed to manage hypertension, the MIND diet was specifically formulated to target the neuropathological mechanisms underlying AD. This DP places particular emphasis on the consumption of foods with neuroprotective properties, especially berries, leafy green vegetables, nuts, and olive oil. Rather than recommending fruits in general, the MIND diet specifically prioritizes berries such as strawberries and blueberries because they are rich in polyphenols like anthocyanins and resveratrol. These bioactive components exert neuroprotective effects through multiple molecular pathways, including the suppression of Aβ generation, the reduction of tau protein hyperphosphorylation, the enhancement of insulin signaling, the attenuation of oxidative stress, and the alleviation of neuroinflammation [31]. Furthermore, the MIND diet underscores the importance of consuming leafy greens such as spinach and kale, which are rich in folate. Folate participates in homocysteine metabolism, and folate supplementation has been demonstrated to reduce plasma homocysteine levels, improve cognitive performance, and inhibit the expression of the pro-inflammatory cytokine TNF-α [32]. Elevated plasma homocysteine is a risk factor for AD [33]. Nuts and olive oil, recognized as key dietary sources of vitamin E, contribute to neuronal protection through antioxidant mechanisms [34]. Studies have consistently shown an inverse association between vitamin E intake and dementia risk. Moreover, AD patients commonly exhibit lower serum vitamin E concentrations compared to healthy controls, further indicating its potential role in preventing cognitive decline [35,36]. Olive oil has also been shown to reduce total Aβ and tau protein in the brain and improve cognitive function [37,38]. Overall, the MIND diet exerts its benefits through the synergistic interaction of multiple mechanisms, offering a viable nutritional intervention to delay the progression of AD pathology.

#### 2.1.4 Western diet

Among the various DPs linked to the risk of AD, the Western diet (WD) has garnered the most extensive attention. The WD is a modern nutritional paradigm centered on ultra-processed foods (UPFs), which are rich in saturated fat, sugar, salt, and cholesterol, while severely lacking dietary fiber, whole grains, and beneficial unsaturated fatty acids [39]. In Western countries, UPFs are convenient and readily available food choices. For instance, in the United States, UPFs contribute approximately 58% of total energy intake and 89% of added sugar consumption [40].

While large-scale epidemiological studies suggest a potential association between UPFs intake and cognitive decline, a direct causal relationship with dementia has not yet been established. A prospective cohort study involving over 860,000 participants found that the high UPFs intake group had a 44% higher risk of all-cause dementia compared to the lowest intake group. This risk exhibited a dose-dependent relationship, with the difference being statistically significant only in the "high-intake" group; no statistical association was observed in the "moderate-intake" group [41]. However, this conclusion has not been consistently replicated. A separate meta-analysis reported a 17% increase in the risk of cognitive impairment associated with UPF intake but found no statistically significant association with AD or clinically diagnosed dementia [42]. Current evidence suggests that UPFs consumption may be more closely related to the early stages of cognitive decline rather than directly causing dementia. Further research is needed to clarify its long-term impacts.

The impact of the WD on neurological risks is supported by profound biological mechanisms. The WD, characterized by its high energy density and glycemic index [43], promotes weight gain, which may trigger a series of health issues associated with metabolic disorders and inflammation. Specific components of the WD, such as cholesterol, saturated fatty acids, and unsaturated fatty acids, are directly linked to inflammatory responses that activate the immune system [44]. Additionally, advanced glycation end products (AGEs) generated during processing, along with other RAGE ligands like Aβ, aggravate neuronal damage by triggering oxidative stress and inflammation [45]. Compared to the MedDiet, the WD markedly​ alters the presynaptic proteome of the cerebral cortex, an alteration closely associated with neurodegenerative diseases and cognitive decline [46]. In AD mouse models, the WD elevates brain metabolism and adaptive immune responses while promoting T-cell infiltration [47]. Collectively, these diet-driven pathological alterations create favorable conditions for AD onset and progression.

#### 2.1.5 MODERN diet

The Machine learning-assisted Optimizing Dietary intERvention against demeNtia risk (MODERN) diet is a novel DP developed based on long-term follow-up data from 185,012 participants in the UK Biobank. Unlike traditional methods relying on empirical judgment in food selection, this diet utilizes advanced machine-learning algorithms to intelligently screen from massive food data and identify the food combinations most effective in reducing dementia risk. The research team pinpointed seven key foods: green leafy vegetables, berries, citrus fruits, potatoes, eggs, poultry, and olive oil. The MODERN diet encourages increased intake of olive oil; recommends moderate consumption of green leafy vegetables, berries, citrus fruits, potatoes, eggs, and poultry; and advocates for the strict restriction of sweetened beverages [48]. While it shares some similarities with the traditional MIND diet, particularly in its recommendation of berries and olive oil, the MODERN diet has been shown to provide greater protective benefits. A 36% reduction in the risk of dementia was observed among individuals with long-term adherence to the MODERN diet over a decade of follow-up. Importantly, the benefits of the MODERN diet are not limited to neuroprotection. It is also linked to a lower risk of various other conditions, including mental and behavioral disorders such as anxiety and depression, and it reduces overall mortality [48].

The potential mechanisms underlying the neuroprotective effects of the MODERN diet are multifaceted. Green leafy vegetables, berries, citrus fruits, and olive oil are rich sources of antioxidants, including polyphenols, vitamin C, and vitamin E, which have been shown to suppress oxidative stress and inflammation. A distinctive feature of the MODERN diet is the deliberate inclusion of potatoes and eggs. Potatoes are abundant in starch, protein, polyphenols, and vitamin B6, exerting antioxidant, anti-inflammatory, and cholesterol-lowering effects [49]. Meanwhile, choline from eggs serves as a precursor for acetylcholine synthesis, a neurotransmitter involved in memory and learning, underscoring the importance of adequate dietary choline intake for cognitive function [50,51]. Eggs also supply certain unsaturated fatty acids like Docosahexaenoic Acid, which are important for brain development and plasticity that has been linked to a lower dementia risk [52,53]. Compared to the MIND diet, the MODERN diet places more stringent restrictions on sweetened beverage consumption. A prospective cohort study based on the UK Biobank reported that daily intake of more than two servings of sugar-sweetened beverages or artificially sweetened beverages was significantly associated with an increased risk of AD, a relationship that may be partially mediated by elevations in blood pressure and the development of insulin resistance [54,55]. Utilizing multi-omics data from the UK Biobank, this research also found that individuals adhering to this DP exhibited better preservation of frontal and temporal cortical thickness and greater white matter structural integrity. Moreover, levels of the inflammatory markers such as Glial Fibrillary Acidic Protein and Interleukin-2 were decreased [48].

### 2.2 Eastern DPs

**Fig. 1:**
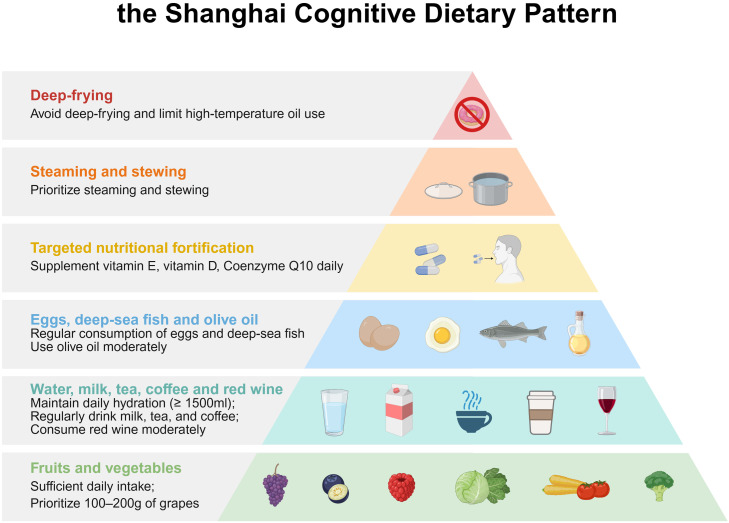
The food Pyramid of the Shanghai Cognitive Dietary Pattern. Graphical elements were applied from BioRender (https://www.biorender.com). The pyramid of this DP systematically integrates food composition, beverage selection, culinary strategies, and nutrient supplementation protocols, presenting its nutritional architecture in a multidimensional manner.

At present, there is a notable research gap regarding DPs for cognitive health in East Asian populations. To address this gap, the researchers from Shanghai Sixth People's Hospital have for the first time proposed and systematically defined a culturally adapted, cognitive-protective DP oriented towards middle-aged and elderly populations in China and East Asia, called the Shanghai Cognitive Dietary Pattern (SCDP). Its detailed composition is presented in Fig. 1.

The empirical foundation for the SCDP is established upon research conducted at the Shanghai Sixth People's Hospital, affiliated with the Shanghai Jiao Tong University School of Medicine. This research focused extensively on the Chinese population aged 45 years and older, ensuring that the evidence base is precisely aligned with the metabolic and nutritional profiles of middle-aged and elderly individuals in East Asia. This pattern first emphasizes the intake of fruits and vegetables, recommending a daily consumption of 100–200 g of berries, particularly grapes. The team at Shanghai Sixth People's Hospital conducted cross-sectional and longitudinal analyses of 177 Chinese elderly individuals with Aβ-PET positivity and found that, in patients with early-stage AD, a daily intake of more than 100 g of fruits (such as grapes) was significantly associated with reduced accumulation of Aβ and Tau proteins in the brain, as well as delayed cognitive decline during follow-up [56]. This protective effect may result from polyphenols in fruits, such as anthocyanins and resveratrol in grape skins. These compounds can inhibit Aβ aggregation and plaque formation, reduce the accumulation of hyperphosphorylated Tau protein, decrease acetylcholinesterase activity, and modulate Aβ-induced microglial inflammatory responses, thereby improving cognitive function [57,58]. Secondly, the SCDP provides detailed beverage recommendations. Men are advised to consume green tea regularly, while women are encouraged to drink plain milk and green tea, with moderate coffee consumption advised. Additionally, the SCDP specifies a minimum daily fluid intake of 1500 ml, addressing the limited attention given to the association between hydration status and cognitive function in current research. Our team at Shanghai Sixth People's Hospital also found that daily fluid intake below 1500 ml (especially below 500 ml) increased the risk of cognitive decline, while consumption of green tea, coffee, or milk was associated with a reduced incidence of cognitive impairment. Notably, among those existing Aβ deposition, long-term consumption of plain milk or green tea may help improve AD pathology [59]. Furthermore, the SCDP emphasizes the direct supplementation of specific nutrients, including the daily intake of B-complex vitamins, vitamin D, and coenzyme Q10. Cross-sectional studies involving middle-aged and elderly Chinese populations have shown that daily supplementation with B vitamins, vitamin D, and coenzyme Q10 is associated with a reduced risk of cognitive impairment in cognitively normal individuals. In addition,​ daily vitamin D supplementation lowers the risk of progression from MCI to AD [60]. Finally, this DP uniquely incorporates cooking methods into the its recommendations. It emphasizes the use of low-temperature, low-oil moist-heat cooking techniques (e.g., steaming and simmering), while avoiding frying and other high-temperature processing methods. Evidence indicates that moist-heat cooking (e.g., steaming and stewing) reduces AGE formation more effectively than dry-heat methods (e.g., baking and frying), thereby lowering overall dietary AGE exposure [61,62]. Given the significant impact of cooking methods on AGE generation, this mechanism provides a theoretical basis for the SCDP's recommendation of moist-heat cooking methods and its opposition to high-temperature frying.

The SCDP guidelines are grounded in empirical data from the aforementioned large-scale dietary study. They are further enhanced by incorporating several key nutrients with established neuroprotective properties, such as n-3 polyunsaturated fatty acids and choline, thereby rendering the framework more comprehensive. Specifically, the model encourages adequate consumption of eggs​ and moderate intake of deep-sea fish and olive oil, as foods rich in these neuroprotective components. A meta-analysis of 21 cohort studies indicates that higher consumption of fish rich in n-3 polyunsaturated fatty acids, particularly docosahexaenoic acid, is significantly associated with a reduced risk of AD [63]. In the context of East Asian DPs, deep-sea fish is specifically emphasized as a primary source of n-3 polyunsaturated fatty acids. Moderate intake of olive oil also holds significant neuroprotective benefits. Olive oil, particularly extra virgin olive oil, is a high-quality source of n-3 polyunsaturated fatty acids. It has been shown to reduce AD risk and, when used as a supplement, to enhance cognitive function in Alzheimer's patients more effectively than other vegetable oils [64]. This effect may be attributed to the loss of certain antioxidant and anti-inflammatory compounds during the refining and processing of other oils [65]. Similarly, red wine is also an important dietary source of polyphenols. Considering that the intake of polyphenols in the daily diet of people in East Asia may generally be insufficient, moderate supplementation of red wine can help to more comprehensively exert the neuroprotective effects of polyphenols. The polyphenols in red wine, such as resveratrol, can exert synergistic effects through multiple pathways. These include inhibiting Aβ aggregation and toxicity, reducing excessive Tau phosphorylation, activating autophagy, improving mitochondrial function, and alleviating brain insulin resistance [66]. Furthermore, multiple prospective cohort studies have confirmed an inverse association between weekly egg consumption and AD risk, with this relationship partly mediated by dietary choline [67,68]. Bioactive components in eggs, such as choline and phosphatidylcholine, may contribute to cognitive preservation and a reduced risk of dementia. These benefits are mediated by maintaining phosphatidylcholine homeostasis in the brain, modulating gene methylation patterns, and alleviating neuroinflammation and oxidative stress [[69], [70], [71]].

Culturally, the SCDP tailors evidence-based principles to the regional context. Certain traditional features, including high sodium intake from pickled vegetables and over-reliance on refined staples, require selective adjustment. To support real-world uptake, the SCDP integrates culturally tailored practices. Natural flavor enhancers such as scallions, ginger, and garlic are promoted to partially replace salt-rich condiments. Cooking methods shift toward steaming, boiling, and stewing, with reduced stir-frying to limit oil use and nutrient loss. While rapeseed oil and freshwater fish remain common staples, moderate consumption of olive oil and deep-sea fish is encouraged to optimize fatty acid profiles. Gradual replacement of refined staples with blended whole-grain and rice models is recommended over sudden dietary changes. Collectively, these pragmatic modifications balance nutritional requirements with local food culture, supporting long-term adherence while preserving cognitive benefits.

### 2.3 A comparison of eastern and western DPs

In the aspect of cognitive health interventions, Western DPs, exemplified by the MIND diet, and East Asian DPs, represented by the SCDP, exhibit differences in their core components and intervention focus. A detailed comparison is presented in Table 2.

**Table 2:** Comparison of eastern and western DPs.

	Western DP (Exemplified by the MIND diet)	Eastern DP (Exemplified by the SCDP)	Core Difference and Localized Value
Fat Source	Primary fat source: olive oil	Olive oil in moderation, relying heavily on fish oil and deep-sea fish	West: Focus on a single high-quality fat. East: Relies on supplements and deep-sea fish.
Vegetables and Fruits	Strict quantification of Green Leafy Vegetables; emphasis on berries	Encourages more fruits, especially grapes; no mandatory vegetable quantification	West: Focus on specific types and raw consumption. East: Emphasis on cooked food, recommending local fruits like grapes.
Protein Source	Fish and poultry as primary protein sources	reliance on eggs and deep-sea fish	East: Emphasizes the consumption of eggs and deep-sea fish.
Cooking Method	Encourages raw and low-temperature Cooking; limits fast-food frying	Advises steaming and stewing; minimizes frying; mostly cooked food	East: Prioritizes healthy cooked food and reshapes traditional Asian culinary habits.
Supportive Intervention	Only restricts sugary beverages	Includes habit items like water, green tea, coffee, and red wine	East: Covers a wider range of lifestyle habits, especially daily water intake.
Nutritional Supplements	No explicit supplement recommendations	Daily supplementation of B vitamins, vitamin D, and coenzyme Q10	East: Favors direct supplementation to address potential nutrient deficiencies.

MIND, Mediterranean-DASH Intervention for Neurodegenerative Delay; SCDP, the Shanghai Cognitive Dietary Pattern.

Regarding fat sources, the Western DP is centered on extra virgin olive oil, emphasizing the intake of monounsaturated fatty acids. In contrast, the East Asian DP recommends moderate olive oil consumption but relies more heavily on deep-sea fish and other aquatic products to provide n-3 polyunsaturated fatty acids, aiming to achieve a balanced fatty acid profile through dietary supplementation. This dietary advice is also informed by geographical differences and resource availability. The Mediterranean climate in Europe supports olive cultivation and animal husbandry, which gradually shaped a dietary structure based on olive oil, dairy, and meat. Conversely, regions like the Yangtze River Basin and southwestern China are major producers of rapeseed oil, reinforcing the predominance of plant-based oils in East Asian diets. Moreover, many East Asian regions have relatively easy access to marine products. For instance, the estuarine environment of the Yangtze River Delta offers abundant aquatic resources, forming a diet in which seafood plays a central role.

In terms of fruit and vegetable consumption, notable cultural differences exist between Eastern and Western DPs. Western DPs emphasize specific amounts of leafy vegetables and encourage raw or low-temperature cooking. In contrast, the East Asian DP imposes fewer quantitative restrictions but prioritizes healthier cooking methods such as steaming and stewing. This approach modifies the common practice of high-temperature stir-frying in Asian cuisines, focusing on reducing local dietary risks associated with cooking. Furthermore, typical Eastern diets often include locally cultivated traditional fruits, such as grapes, whereas Western dietary recommendations emphasize anthocyanin-rich berries, particularly blueberries and raspberries.

Lastly, the scope of dietary interventions in Eastern patterns is more comprehensive. Beyond food choices, they explicitly incorporate daily lifestyle factors, including adequate hydration, regular tea consumption, moderate coffee intake, and appropriate use of dietary supplements. Among these, the emphasis on adequate hydration represents a nutritional aspect that has received limited research attention. Furthermore, tea drinking reflects the profound influence of cultural traditions on dietary practices. Originating in Asia, tea consumption is deeply embedded in the daily routines of most Chinese and other East Asian populations. Therefore, in studying East Asian DPs, it is essential to integrate tea culture as a key cultural dimension into a comprehensive analytical framework.

Both the MIND diet and the SCDP aim to promote brain health, but they differ significantly in their approaches. The MIND diet is well-supported by evidence and features a clear framework that encourages the consumption of ten food groups while restricting five. However, its reliance on specific components such as olive oil and berries may limit its generalizability. In contrast, the SCDP emphasizes precision and local adaptation. It provides quantitative recommendations, including exact gram amounts and gender-specific distinctions, while highlighting cooking methods such as steaming that are well aligned with Asian dietary customs. Although its long-term efficacy requires further empirical validation, the SCDP stands out for its personalized and culturally tailored approach, offering a more targeted strategy for brain health management in Asian populations.

## 3 Dietary intervention strategies

Dietary intervention for AD is a multidimensional and dynamic process that operates across both population and individual scales. Given the complexity of AD pathogenesis, dietary optimization alone is insufficient; therefore, dietary optimization must be combined with other lifestyle and multimodal interventions. Particular emphasis should be placed on implementing forward-looking preventive strategies during the critical middle-aged window, while simultaneously accounting for regional dietary differences between Eastern and Western populations, ultimately achieving targeted prevention and treatment of AD.

### 3.1 From population adaptation to individual precision

At the population level, current research on neuroprotective DPs predominantly relies on Western biodatabases. Given the distinct variations in dietary habits and physiological profiles, future work must transition from merely validating Western-centric models to developing regionally adapted nutritional frameworks that promote long-term adherence.

Furthermore, this review does not provide a detailed assessment of the Ketogenic Diet (KD), primarily because its clinical utility is characterized as a short-term, clinically supervised therapeutic intervention rather than the sustainable, lifelong dietary framework prioritized in our analysis. Although the KD exhibits robust acute neuroprotective potential, it functions as a stringent clinical tool, distinct from the preventive strategies central to this discussion.

Transitioning from population to the individual level, precision nutrition aims to refine broad dietary recommendations by incorporating individual biological specificity.​ The core of the personalized dietary intervention strategy is the integration of an individual's multi-omics data, including genomics, metabolomics, and other relevant datasets, to design targeted dietary interventions [72]. For example, gut microbiome characteristics, such as gene richness and specific bacterial abundance, have been identified as novel predictive markers and potential targets for intervention. Beyond this, effective strategies must account for additional individual variations, including genetic background, AD disease stages, specific life stages, gender, and age. This layered approach ensures that dietary interventions are not only mechanistically justified but also sufficiently flexible for real-world implementation.

### 3.2 Dietary interventions in combination with other measures

The complex pathophysiology of AD dictates that single intervention strategies often yield limited effects. Accordingly, a highly promising approach is to combine dietary interventions with other measures, thereby forging synergistic intervention strategies. This synergistic effect is supported by emerging theoretical frameworks [73]. Rao et al. conceptualized AD's complex pathology as "network insufficiency." This perspective extends beyond traditional Aβ/Tau hypotheses to characterize AD as a systemic network failure triggered by over 36 risk factors, including diet, toxins, and infections [74]. This concept underscores AD's multifactorial etiology.

**Fig. 2:**
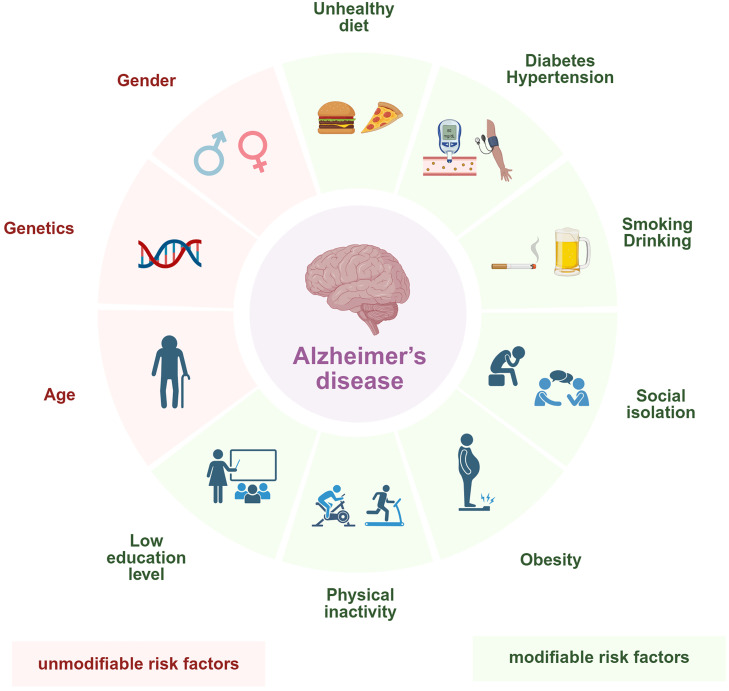
Risk factors for AD. Graphical elements were applied from BioRender (https://www.biorender.com). The major risk factors for AD are classified into two main categories: unmodifiable risk factors and modifiable risk factors. Unmodifiable factors, which cannot be changed, include age, genetics, and gender. Modifiable factors, which lifestyle changes or medical interventions can influence, include unhealthy diet, diabetes and hypertension, smoking and drinking, social isolation, obesity, physical inactivity, and low education level. Modifying these factors through lifestyle changes can be a preventive or interventional measure for AD.

As depicted in Fig. 2, the majority of risk factors are modifiable, including unhealthy diet, hypertension, smoking, and physical inactivity. This is in sharp contrast to the unmodifiable risk factors, such as sex, genotype, and age. Therefore, interventions targeting these modifiable factors are crucial. Combining a healthy diet with other lifestyle interventions, including regular physical activity and smoking cessation, exerts​ synergistic effects that delay cognitive decline more effectively than single interventions [75,76]. The Finnish Geriatric Intervention Study confirmed that a two-year multifactorial intervention targeting high-risk older adults, such as diet, exercise, cognitive training, and vascular risk monitoring, can effectively improve cognitive function [77]. Consequently, integrated multimodal interventions are imperative for maximizing overall efficacy, offering substantial advantages over single interventions.

## 4 Conclusions and perspectives

AD imposes a substantial clinical burden on patients and families. Compounding this burden is the limited efficacy of current pharmacotherapies, which underscores the urgent need for non-pharmacological alternatives. Given the marked divergence in dietary habits between Eastern and Western populations, this review proposes the SCDP as a tailored nutritional strategy and compares traditional Western DPs with the SCDP. Developed for Chinese adults aged 45 years and older, the SCDP is informed by large-scale cross-sectional analyses identifying key neuroprotective components spanning beverages (e.g., tea, coffee), essential micronutrients (e.g., B vitamins, coenzyme Q10), and polyphenol-rich foods (e.g., berries). Furthermore, the framework incorporates specific food sources rich in choline, n-3 polyunsaturated fatty acids, and other critical nutrients, while advocating a shift toward cooking methods such as steaming and stewing. In conclusion, currunt Western dietary models often fail to accommodate the distinct physiological needs​ of Asian populations. Therefore, effective nutritional interventions must be tailored to regional dietary habits and individual metabolic profiles. We assert that optimizing cognitive health requires selecting appropriate and sustainable dietary patterns tailored to specific populations, rather than adhering to a universal standard. Advancing dietary interventions from population-level adaptation to individual precision, coupled with multimodal lifestyle management, produces concrete benefits for both the prevention and management of AD.

Despite the promise of these interventions, significant challenges hinder their integration into routine clinical practice. Current evidence is largely based on animal studies and observational studies, and high-quality randomized controlled trials are still scarce. Major limitations include dependence on self-reported dietary data prone to recall bias, and generally low adherence to strict diet plans. Future investigations must prioritize high-quality randomized controlled trials to address these gaps. Multi-omics approaches, such as metabolomics, can refine personalized predictive models but raise ethical issues concerning data privacy and informed consent. Other challenges include the substantial resources required to implement highly individualized dietary interventions, while their cost-effectiveness is not well established. Ultimately, ensuring the long-term feasibility and adherence of these dietary strategies is essential for realizing their real-world clinical benefits.

## Declarations

**Ethics approval and consent to participate:** Not applicable.

**Consent for publication:** Not applicable.

**Availability of data and materials:** Not applicable

## Declaration of generative AI and AI-assisted technologies in the writing process

During the preparation of this work, the authors did not use any generative AI or AI-assisted technologies.

## Funding

This work was supported by Scientific Research Project of Shanghai Municipal Health Commission (202340080), the Basic Research Project of Shanghai Sixth People's Hospital (General Cultivation Project, ynms202207) and 10.13039/501100001809National Natural Science Foundation of China (82171198).

## CRediT authorship contribution statement

**Wanlu Jiang:** Writing – review & editing, Writing – original draft, Methodology, Formal analysis, Conceptualization. **Yijia Lin:** Writing – review & editing, Writing – original draft, Methodology, Formal analysis, Conceptualization. **Qihao Guo:** Writing – review & editing, Supervision, Methodology, Conceptualization. **Ya Miao:** Conceptualization, Methodology, Writing – review & editing, Supervision.

## Declaration of competing interest

The authors declare that they have no known competing financial interests or personal relationships that could have appeared to influence the work reported in this paper.
